# Prevalence and staging of non-alcoholic fatty liver disease among patients with heart failure with preserved ejection fraction

**DOI:** 10.1038/s41598-020-69013-y

**Published:** 2020-07-24

**Authors:** Alexandria Miller, Jennifer McNamara, Scott L. Hummel, Matthew C. Konerman, Monica A. Tincopa

**Affiliations:** 1 Division of Gastroenterology and Hepatology, Department of Internal Medicine, University of Michigan, 1500 East Medical Center Drive, Ann Arbor, MI 48109 USA; 2 Department of Internal Medicine, University of Michigan, Ann Arbor, MI USA; 3 Division of Cardiovascular Medicine, Department of Internal Medicine, University of Michigan, Ann Arbor, MI USA; 4 Ann Arbor Veterans Affairs Health System, Ann Arbor, MI USA

**Keywords:** Cardiology, Gastroenterology

## Abstract

Insulin resistance and altered energy metabolism is common in non-alcoholic fatty liver disease (NAFLD) and appears to also be associated with myocardial dysfunction. We aimed to evaluate prevalence, staging and clinical features correlated with NAFLD among patients with heart failure with preserved ejection fraction (HFpEF). Adults with HFpEF were prospectively enrolled. Demographic and clinical data were collected. NAFLD was defined based on liver biopsy, abdominal imaging or ICD-coding and the absence of other liver diseases. Descriptive, bivariate and multivariable analyses were performed. 181 patients were analyzed. The median age was 70 with 89% white, 59% female, median BMI 35.1, and 48% with diabetes. NAFLD was present in 27% of the full cohort and 50% of those with imaging. In patients with imaging, multivariable analysis identified diabetes (OR 3.38, 95% CI 1.29–8.88) and BMI (OR 1.11, 95% CI 1.04–1.19) as independent correlates of NAFLD. 54% of NAFLD patients had a NAFLD fibrosis score consistent with advanced fibrosis. Cirrhosis was present in 6.6% of patients overall and 11.5% with imaging. NAFLD patients had a higher frequency of advanced heart failure (75% vs 55%, p 0.01). NAFLD has a two-fold higher prevalence in HFpEF compared to the general population and is independently associated with BMI and diabetes. Patients with HFpEF and NAFLD also appeared to have more advanced fibrosis including cirrhosis suggesting a potential synergistic effect of cardiac dysfunction on fibrosis risk in NAFLD. This data supports consideration for evaluation of underlying liver disease in HFpEF patients.

## Introduction

Heart failure with preserved ejection fraction (HFpEF) and non-alcoholic fatty liver disease (NAFLD) are two disease entities that have been growing in prevalence over the last decade. HFpEF is now more prevalent than heart failure with reduced ejection fraction (HFrEF), and NAFLD is estimated to be present in 25–30% of the adult United States population^1,2^. This high prevalence likely relates to the association of these entities with obesity-related conditions including hypertension, insulin resistance, and dyslipidemia^1,3^. While multiple studies have linked NAFLD with a higher risk of cardiovascular disease and cardiovascular mortality, particularly in terms of coronary artery disease, the relationship between NAFLD and heart failure is less clear. Emerging data has outlined an association between NAFLD and changes in myocardial structure and diastolic dysfunction^2,4,5^. The relationship between NAFLD and HFpEF remains unclear and requires further investigation in order to inform clinical management of this population.

In this study, we aimed to evaluate the prevalence and correlates of NAFLD among a cohort of patients with HFpEF. In addition, we aimed to evaluate disease stage among this unique patient population. We hypothesized that NAFLD is prevalent at higher rates in HFpEF than in the general population, and that individuals with both HFpEF and NAFLD may have more advanced disease stage due to synergistic effects of metabolic risk factors on progression of disease.

## Methods

### Patient population

Patients who met diagnostic criteria for HFpEF and who were followed as part of the University of Michigan HFpEF outpatient clinic were prospectively enrolled into a research database. The diagnosis of HFpEF was ascertained by board certified cardiologists with expertise in this disease entity (S.L.H. or M.C.K.). HFpEF was defined according to the criteria established by the 2016 European Society of Cardiology guidelines^6^. In brief, this definition requires symptoms and signs of heart failure (elevated natriuretic peptide and either relevant structural heart disease or diastolic dysfunction) among patients with ejection fraction ≥ 50%. The database encompasses patients presenting from February 2014 to February 2018.

### Data collection

At the time of enrollment, demographics, vital signs, medications, and comorbid conditions were recorded. New York Heart Association (NYHA) classification was documented as an assessment of heart failure severity. Dyslipidemia was defined based on use of statin or cholesterol lowering medications or ICD-9 or 10 codes. Additional data not entered at time of initial enrollment were abstracted retrospectively via electronic chart review. Laboratory data including complete blood cell counts, lipids, kidney function, hepatic panel, international normalized ratio (INR), hemoglobin A1c, and pro-brain natriuretic peptide (BNP) were abstracted if obtained during routine clinical care within two years of the initial clinic encounter. Echocardiograms (ECHO) obtained within 2 years of the clinic encounter were reviewed and measurements of chamber size, wall thickness, diastolic function, and right ventricular function were recorded. The study was approved by the University of Michigan Institutional Review Board (IRB). Given use of de-identified patient data, the IRB granted exception for need of individual patient consent for the retrospective analysis. All patients provided informed consent to initially be included in the HFpEF registry. All methods were performed in accordance with the relevant guidelines and regulations.

### Assessment of liver disease

The diagnosis of NAFLD was ascertained using a combination of natural language processing program (University of Michigan EMERSE^7^) and manual chart review by a board certified hepatologist (M.A.T.) with specific expertise in NAFLD^7^. A patient was classified as meeting criteria for NAFLD if they had any of the following in the absence of other causes of liver disease: evidence of steatosis or steatohepatitis on liver biopsy; imaging [including ultrasound (US), computed tomography (CT) scan or magnetic resonance imaging (MRI)] noting hepatic steatosis; a clinical diagnosis code (ICD-9 or 10) for NAFLD or non-alcoholic steatohepatitis (NASH); or a combination of the above. Given clinical practice patterns, very few patients had liver biopsy performed and thus it was not possible to definitively distinguish NAFLD vs NASH among the entire cohort. Patients were also assessed for a history of significant alcohol use (defined as > 21 drinks per week in men and > 14 drinks per week in women or clinical diagnosis of alcohol use disorder) based on chart review. In addition, charts were reviewed for other causes of chronic liver disease including viral hepatitis, autoimmune liver disease or hereditary forms of liver disease. The presence of cirrhosis was determined based on results of imaging or ICD-9 or 10 codes. To assess stage of liver disease, a Fibrosis-4 (FIB-4) Index and NAFLD Fibrosis Score (NFS) were calculated for each patient, both of which have been used in heart failure patients^8–10^. Advanced fibrosis was defined using the validated cut-offs of > 0.675 for NFS and > 3.25 for FIB-4^11,12^. For multivariable analysis of correlates of advanced fibrosis, we used results of NFS and those meeting criteria for cirrhosis based on imaging results or ICD-9 or 10 codes.

### Statistical analysis

Given the sample size, data were evaluated for normality and summarized with median and interquartile range (IQR) values or percentages as appropriate. The Kruskal–Wallis rank-sum test, Chi-square and Fisher exact and t-testis were used to compare differences in clinical variables between patients with and without NAFLD or advanced fibrosis. Univariate and multivariable logistic regression were used to identify significant predictors of NAFLD and advanced fibrosis. Candidate covariates were assessed for inclusion into the multivariable model based on p values < 0.10 in the univariate analysis and biologic plausibility. We performed a subanalysis among patients with abdominal imaging as this subset represents individuals in whom some sort of screening for NAFLD was performed and thus could more definitely categorize patients for the presence or absence of NAFLD. p values < 0.05 were considered statistically significant. All statistical analyses were performed in SAS (Version 9.4, Copyright 2016 by SAS Institute Inc., Cary, NC, USA).

## Results

### Patient population

All 181 patients enrolled in the HFpEF clinical database were analyzed. Baseline characteristics are shown in Table 1. The cohort consisted primarily of advanced age, white females with obesity. The vast majority had hypertension and dyslipidemia. Regarding heart failure severity, 61% had severe heart failure symptoms (NYHA classification III–IV). Diastolic dysfunction of grade ≥ 2 diastolic dysfunction on echocardiography was noted in over 40% of patients.

**Table 1:** Baseline characteristics of patients by NAFLD status.

Variable	Overall (n = 181)	NAFLD (n = 49)	No NAFLD (n = 133)	p value
**Demographics**				
Age	70 (59–77)	69 (57–75)	70 (60–78)	0.34
Female sex	75 (41%)	31 (63%)	75 (57%)	0.43
Weight (kg)	95.7 (79.4–115)	108 (90.1–121)	92.7 (76.6–110)	**< 0.001**
BMI (kg/m^2^)	35.1 (28.2–42.1)	37.9 (33.3–45.1)	33.6 (27.6–40.4)	**0.004**
**Clinical characteristics**				
Hypertension	147 (81%)	41 (84%)	106 (80%)	0.61
Coronary artery disease	75 (41%)	21 (43%)	54 (41%)	0.81
Diabetes mellitus	87 (48%)	30 (61%)	57 (43%)	**0.03**
Obesity (BMI > 30 kg/m^2^)	125 (69%)	42 (86%)	83 (63%)	**0.003**
Atrial fibrillation	87 (48%)	25 (51%)	62 (47%)	0.63
Dyslipidemia	119 (66%)	36 (73%)	83 (63%)	0.18
Statin use	123 (68%)	37 (76%)	86 (65%)	0.18
Chronic kidney disease (GFR < 60)	96 (53%)	27 (55%)	69 (52%)	0.73
**Heart failure severity**				
NYHA class	n = 176	n = 49	n = 127	**0.01**
I–II	69 (39%)	12 (25%)	51 (45%)	
III–IV	107 (61%)	37 (75%)	76 (55%)	
BNP (pg/mL)	117 (52.0–260)	95.5 (29.0–224)	126 (58.0–263)	0.20
**Echocardiography**				
Ejection fraction (n = 172)	60 (60–65)	60 (60–65)	60 (60–65)	0.83
IVS (n = 160)	10 (9.0–12)	11 (9–12)	10 (9–12)	0.19
Left atrial diameter (n = 168)	46 (40–52)	46 (41–50)	46 (39–53)	0.78
RVSP (n = 116)	47 (35–58)	47 (33–54)	47 (36–59)	0.42
E/A ratio (n = 104)	1.2 (0.8–1.9)	1.1 (0.8–1.8)	1.2 (0.9–1.9)	0.40
Diastolic dysfunction (n = 91)				
Grade 0–1 or indeterminate	53 (58%)	14 (56%)	39 (59%)	0.79
Grade ≥ 2	38 (42%)	11 (44%)	27 (41%)	
RV systolic dysfunction (n = 166)	23 (14%)	4 (9%)	19 (16%)	0.26
**Metabolic evaluation**				
Total cholesterol (n = 156)	156 (124–184)	139 (112–176)	159 (128–186)	0.08
Triglycerides (n = 156)	124 (88–170)	122 (92–177)	126 (86–169)	0.84
HDL (n = 156)	46 (38–59)	43 (36–53)	48 (39–61)	0.12
LDL (n = 156)	76 (60–97)	70 (46–91)	82 (64–100)	**0.02**
A1c (n = 131)	6.2 (5.7–7.6)	6.4 (5.8–8.5)	6.2 (5.7–7.2)	0.19
**Hepatic evaluation**				
Platelets (n = 178)	215 (176–268)	217 (187–273)	214 (167–268)	0.44
AST (n = 179)	26 (20–31)	25 (21–31)	26 (20–31)	0.98
ALT (n = 179)	23 (17–32)	24 (17–32)	23 (17–31)	0.91
INR (n = 160)	1.1 (1.0–1.4)	1.1 (1.0–1.4)	1.1 (1.0–1.4)	0.94
Total bilirubin (n = 178)	0.5 (0.4–0.8)	0.5 (0.4–0.8)	0.5 (0.4–0.7)	0.79
Albumin (n = 178)	4.1 (3.9–4.4)	4.1 (3.8–4.5)	4.1 (3.9–4.4)	0.96
FIB-4 (n = 160)	1.6 (1.2–2.5)	1.5 (1.0–2.4)	1.7 (1.2–2.6)	0.33
NFS (n = 159)	0.33 (− 0.72 to 1.4)	0.54 (− 0.72 to 1.59)	0.22 (− 0.74 to 1.24)	0.16
Cirrhosis by imaging (n = 96)	12 (6.6%)	6 (12%)	6 (4.6%)	0.06

Bolded values indicate results that are statistically significant

*NYHA* New York Heart Association, *BNP* b-type natriuretic peptide, *IVS* interventricular septum thickness, *RVSP* right ventricular systolic pressure, *NFS* NAFLD Fibrosis Score, *LDL* low density lipoprotein cholesterol, *HDL* high density lipoprotein cholesterol.

### Prevalence and correlates of NAFLD

Overall, 49 patients (27%) met criteria for NAFLD and 12 had imaging consistent with cirrhosis. Patients with NAFLD had higher BMI (p = 0.004) and more frequently had diabetes (p = 0.03) and NYHA class III–IV heart failure (p = 0.01). Of note, low density lipoprotein (LDL) cholesterol was lower in the patients with NAFLD (p = 0.02) though a greater percentage of NAFLD patients were receiving statin therapy (p = 0.18). On multivariable analysis, only LDL was associated with risk of NAFLD within the overall cohort (OR 0.98, 95% CI 0.97–0.99, p = 0.04, Supplemental Table 1).

Analysis of only patients with prior abdominal imaging (N = 96) noted a higher prevalence of NAFLD (50%) and cirrhosis (11.5%) (Table 2). Among patients with imaging, NAFLD patients again had higher BMI, rates of diabetes and more severe heart failure. They also had higher median septal thickness (11 vs. 10 mm, p = 0.01). On multivariable analysis, BMI (OR 1.11 per unit kg/m^2^, 95% CI 1.04–1.18, p = 0.001) and diabetes (OR 3.38, 95% CI 1.29–8.88, p = 0.01) were associated with an increased risk of NAFLD (Table 3). Of note, patients who underwent imaging were less likely to have anemia and had lower measures of right ventricular systolic pressure, B-type natriuretic peptide, total cholesterol, and low-density lipoprotein (Supplemental Table 2).

**Table 2:** Baseline characteristics by NAFLD status among patients with imaging.

Variable	Overall (n = 96)	NAFLD (n = 48)	No NAFLD (n = 48)	p value
**Demographics**				
Age	69 (58–76)	70 (58–76)	69 (60–76)	0.90
Female sex	39 (41%)	31 (65%)	27 (56%)	0.40
Weight (kg)	99.2 (80.6–114)	108 (89.9–120)	89.7 (72.8–108)	**< 0.001**
BMI (kg/m^2^)	35.1 (27.8–41.4)	38.1 (33.3–45.2)	30.2 (26.4–37.7)	**< 0.001**
**Clinical characteristics**				
Hypertension	78 (81%)	40 (83%)	37 (77%)	0.44
Coronary artery disease	39 (41%)	20 (42%)	20 (42%)	1.00
Diabetes mellitus	49 (51%)	30 (62%)	19 (40%)	**0.02**
Obesity (BMI > 30 kg/m^2^)	66 (69%)	41 (85%)	25 (52%)	**< 0.001**
Atrial fibrillation	44 (46%)	24 (50%)	20 (42%)	0.41
Dyslipidemia	66 (69%)	35 (73%)	32 (67%)	0.50
Statin use	67 (70%)	36 (75%)	32 (67%)	0.36
Chronic kidney disease (GFR < 60)	50 (52%)	27 (56%)	23 (48%)	0.41
**Heart failure severity**				
NYHA class	n = 95	n = 48	n = 47	
I–II	34 (36%)	11 (23%)	23 (49%)	NS
III–IV	61 (64%)	37 (77%)	24 (51%)	**0.008**
BNP (pg/mL)	102 (32–199)	89 (26–237)	106 (32–191)	0.78
**Echocardiography**				
Ejection fraction (n = 91)	60 (60–65)	60 (60–65)	64 (60–65)	0.62
IVS (n = 90)	10 (9–12)	11 (9–12)	10 (9–11)	**0.01**
Left atrial diameter (n = 91)	45 (39–50)	46 (41–50)	42 (37–50)	0.24
RVSP (n = 57)	42 (34–54)	47 (33–54)	41 (34–54)	0.50
E/A ratio (n = 59)	1.1 (0.8–1.8)	1.1 (0.8–1.8)	1.2 (0.8–1.8)	0.79
Diastolic dysfunction	n = 49	n = 25	n = 24	
Grade 0–1 or indeterminate	31 (63%)	14 (56%)	17 (74%)	NS
Grade ≥ 2	18 (37%)	11 (44%)	7 (26%)	0.19
RV Systolic Dysfunction (n = 88)	10 (11%)	4 (9%)	6 (14%)	0.50
**Metabolic evaluation**				
Total cholesterol (n = 89)	150 (122–173)	138 (112–176)	154 (130–169)	0.36
Triglycerides (n = 89)	106 (82–158)	116 (91–178)	102 (78–148)	0.20
HDL (n = 89)	45 (36–54)	43 (36–53)	48 (38–62)	0.16
LDL (n = 89)	71 (55–90)	68 (46–92)	75 (58–90)	0.17
A1c (n = 81)	6.3 (5.7–7.6)	6.3 (5.8–8.5)	6.2 (5.6–7.0)	0.11
**Hepatic evaluation**				
Platelets (n = 94)	216 (181–272)	218 (187–273)	209 (154–266)	0.35
AST (n = 95)	26 (20–32)	25 (21–30)	28 (20–33)	0.51
ALT (n = 95)	23 (17–30)	24 (16–31)	21 (17–29)	0.76
INR (n = 89)	1.1 (1.0–1.3)	1.1 (1.0–1.3)	1.1 (1.0–1.3)	0.82
Total bilirubin (n = 95)	0.5 (0.4–0.8)	0.5 (0.4–0.8)	0.5 (0.4–0.8)	0.86
Albumin (n = 95)	4.2 (3.8–4.4)	4.1 (3.8–4.5)	4.2 (3.8–4.4)	0.75
FIB-4 (n = 88)	1.62 (1.08–2.34)	1.46 (0.98–2.33)	1.77 (1.15–2.73)	0.21
NFS (n = 88)	0.32 (− 0.82 to 1.38)	0.54 (− 0.72 to 1.59)	− 0.03 (− 1.25 to 1.04)	0.06
Cirrhosis by imaging	11 (11%)	6 (12%)	5 (10%)	0.74

Bolded values indicate results that are statistically significant

**Table 3:** Multivariable correlates of NAFLD among HFpEF patients: imaging subset.

Variable	Odds ratio	95% CI	p value
Age (per year)	1.02	(0.98–1.07)	0.22
Sex	1.27	(0.47–3.43)	0.48
Diabetes	3.38	(1.29–8.88)	**0.01**
BMI	1.11	(1.04–1.18)	**0.001**
NYHA Class 3 or 4	2.79	(0.98–7.88)	0.05

Bolded values indicate results that are statistically significant

### Prevalence and risk factors for advanced fibrosis or cirrhosis

**Figure 1:**
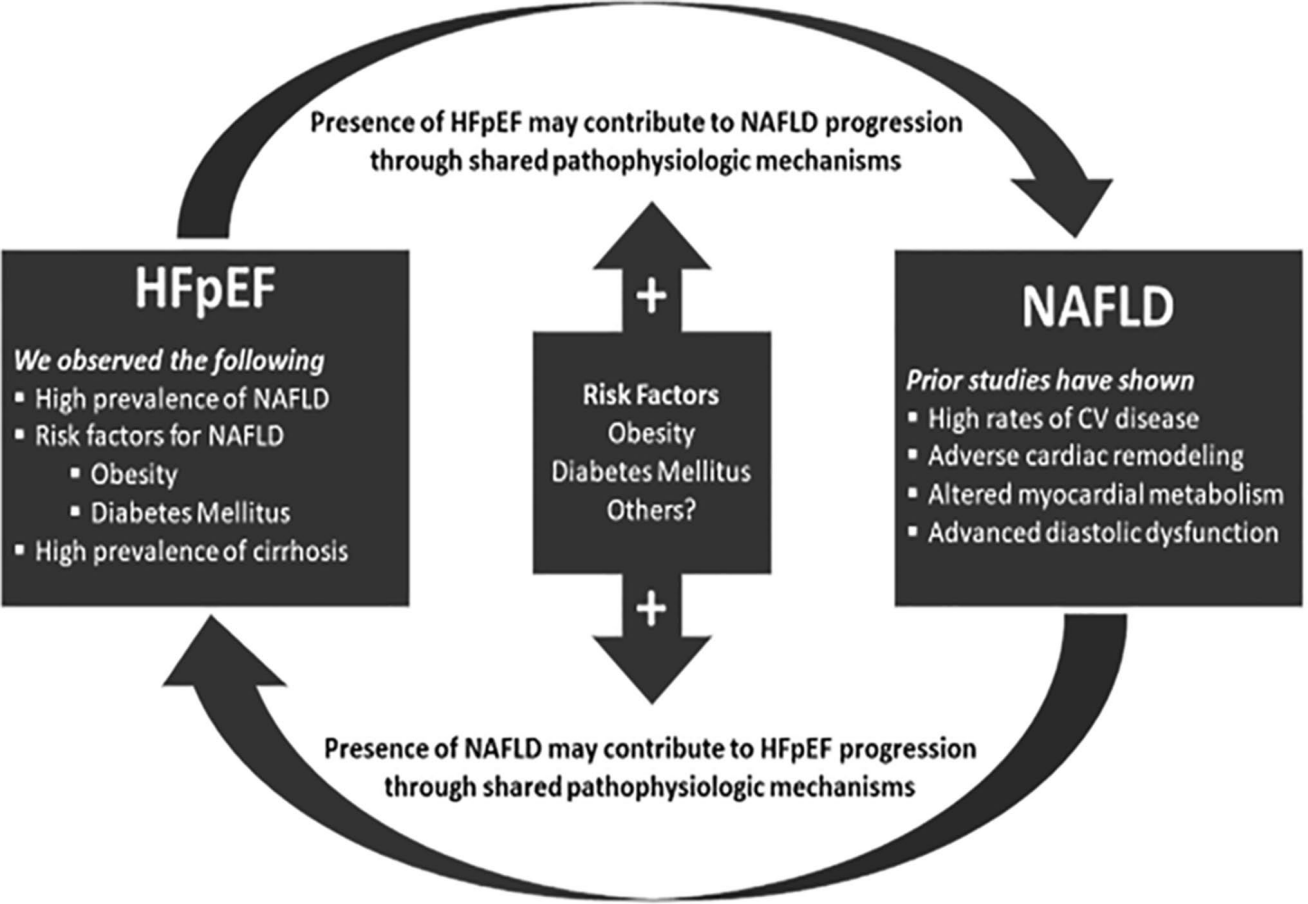
Proposed shared pathophysiologic mechanisms in HFpEF and NAFLD.

A total of 88 patients (48.6%) had advanced fibrosis or cirrhosis by imaging or NFS (Table 4). Among individuals with NAFLD, 54% had NFS consistent with at least advanced fibrosis. Cirrhosis was present in 6.6% of patients overall and 11.5% of patients with imaging. In the overall cohort, patients with advanced fibrosis or cirrhosis were older, had higher BMI, and more likely to have diabetes, atrial fibrillation and chronic kidney disease. On echocardiography, those with advanced fibrosis or cirrhosis also had larger left atrial diameter (47 vs 43) and more commonly had ≥ grade 2 diastolic dysfunction. As expected, these patients also had higher INR and total bilirubin levels. On multivariable analysis, older age, higher BMI and presence of diabetes were all independently associated with advanced fibrosis or cirrhosis. (Table 5; Fig. 1).

**Table 4:** Baseline characteristics of patients with and without cirrhosis or high NFS.

Variable	Advanced fibrosis or cirrhosis (n = 88)	No advanced fibrosis or cirrhosis (n = 93)	p value
**Demographics**			
Age	71.1 (61–79)	66 (56–75)	**0.005**
Male sex	47 (53.4%)	59 (63.4%)	0.17
BMI (kg/m^2^)	37.4 (30.1–44.6)	33.6 (27.5–39.7)	**0.008**
**Clinical characteristics**			
Hypertension	74 (84.1%)	73 (78.5%)	0.33
Coronary artery disease	39 (44.3%)	36 (38.7%)	0.44
Diabetes mellitus	52 (59.1%)	35 (37.6%)	**0.004**
Atrial fibrillation	51 (57.9%)	36 (38.7%)	**0.01**
Dyslipidemia	58 (65.9%)	61 (65.5%)	0.96
Chronic kidney disease	53 (60.2%)	43 (46.2%)	**0.05**
**Heart failure severity**			
NYHA class III–IV	58 (62.3%)	51 (56.0%)	0.07
BNP	143 (64.5–299.5)	102 (34–212)	0.06
**Echocardiography**			
Ejection fraction	60 (60–65)	60 (60–65)	0.77
IVS	11 (10–13)	10 (9–12)	0.06
Left atrial diameter	47 (44–54)	43 (38–49)	**0.001**
RVSP	50 (38–59)	43 (33–58)	0.25
E/A ratio	1.25 (1.0–1.6)	1.1 (0.8–2.0)	0.73
Diastolic dysfunction Grade ≥ 2	21 (53.8%)	17 (32.7%)	**0.04**
RV systolic dysfunction	17 (20.7%)	9 (10.5%)	0.06
**Metabolic evaluation**			
Total cholesterol	153.5 (119.5–176.5)	160 (134–187.5)	0.13
Triglycerides	113 (89.5–162)	129 (86–178)	0.58
HDL	44.5 (36–56.5)	47 (39–60.5)	0.41
LDL	70 (57.5–91.5)	82 (60.5–105)	0.06
A1c	6.3 (5.8–7.6)	6.2 (5.6–7.4)	0.46
**Hepatic evaluation**			
INR	1.1 (1–1.5)	1 (0.97–1.2)	**0.02**
Total bilirubin	0.6 (0.4–0.9)	0.5 (0.3–0.7)	**0.001**

Bolded values indicate results that are statistically significant

**Table 5:** Multivariable correlates of advanced fibrosis or cirrhosis among HFpEF patients.

Variable	Odds ratio	95% CI	p value
Age (per year)	1.07	1.01–1.13	**0.01**
Sex	1.34	0.44–4.03	0.53
BMI	1.07	1.01–1.15	**0.02**
Diabetes	3.93	1.4–10.97	**0.009**
Atrial fibrillation	1.19	0.35–3.98	0.77
CKD	1.18	0.43–3.21	0.73
Left atrial diameter	1.01	0.93–1.09	0.77
Diastolic dysfunction grade ≥ 2	1.03	0.33–3.18	0.95

Bolded values indicate results that are statistically significant

## Discussion

The clinical and public health implications of obesity and metabolic syndrome are continuing to expand with increasing prevalence of chronic conditions stemming from these processes. While individual obesity related conditions have been investigated in detail, there has been less data evaluating concomitant disease processes and how these comorbid conditions interact to impact disease severity and progression. In this study, we highlight the significantly higher prevalence of NAFLD in a well-defined cohort of HFpEF patients and note the more advanced disease stages (both heart failure class and hepatic fibrosis stage) present in this unique patient population.

Prior literature has shown a clear association between NAFLD and overall cardiac dysfunction^4,13–16^. However, there is conflicting data in terms of the relationship between NAFLD and specific patterns of cardiac dysfunction^17–19^. To date, there has been most focus on the relationship between NAFLD and diastolic dysfunction. HFpEF has become a prominent condition in our aging population with high rates of comorbid chronic conditions. Given the nuances in making an accurate diagnosis of HFpEF, evaluation for these concomitant disorders can be challenging. In this well characterized cohort of HFpEF patients, we found that NAFLD was present in 27% of the full cohort and 50% of individuals with prior imaging, and thus prior evaluation for possible NAFLD. This 50% prevalence is twofold higher than the general population. As would be expected, diabetes (OR 3.38) and BMI (OR 1.11) were independently associated with NAFLD among patients with HFpEF. Interestingly, while heart failure severity was associated with higher prevalence of NAFLD on univariate analysis, this did not remain significant on multivariable analysis. No other cardiac parameters on echocardiography, including evidence of diastolic dysfunction or right-sided heart failure, were found to be independently associated with NAFLD either. This high disease prevalence is clinically significant as patients with NAFLD have been shown to have increased mortality compared to matched controls without NAFLD, and given that the presence of NAFLD is associated with a 26% higher health care cost^20,21^. NAFLD patients have also been shown to have more severe metabolic derangements compared to individuals with obesity without NAFLD, making their clinical management more challenging^22^.

In addition to high disease prevalence, in this cohort we also noted an important association between more advanced heart failure and hepatic fibrosis stage among individuals with both HFpEF and NAFLD. In this patient population, 49% of the patients had advanced fibrosis or cirrhosis by imaging or NFS. Among individuals with NAFLD, 54% had NFS consistent with at least advanced fibrosis. Cirrhosis was present in 6.6% of patients overall and 11.5% of patients with imaging. This prevalence of cirrhosis is markedly higher than the cited prevalence of cirrhosis for adults in the United States (0.27%)^23^. Older age (OR 1.07), higher BMI (OR 1.07) and diabetes (OR 3.93) were independently associated with advanced fibrosis or cirrhosis among patients with HFpEF. Again of note, no specific cardiac parameters on echocardiography appeared to be independently associated with more advanced hepatic fibrosis. Prior studies have noted an association between NAFLD and indices of impaired relaxation and increased left-sided chamber pressures on echocardiography^14,17,18,24^. This finding is important given that prior data that has shown that liver disease stage is strongly linked with outcomes among patients with heart failure^9,25^. More advanced liver fibrosis has been associated with volume overload, diastolic dysfunction, impaired exercise capacity, and higher mortality in patients with HFpEF^5,8,9^. NAFLD may be linked to HFpEF progression through its association with increased epicardial fat, particularly in patients with more advanced liver fibrosis^19,24,26^. Epicardial adipose tissue is associated with the secretion of proinflammatory adipokines that can cause atrial and ventricular fibrosis^27^. In addition, epicardial fat and hepatic triglyceride content in NAFLD patients are associated with myocardial insulin resistance and reduced myocardial perfusion. It is well known that patients with heart failure can develop “cardiac cirrhosis” due to chronic hepatic congestion with resultant fibrosis^28^. As such, there is likely a synergistic effect of heart failure with potential for hepatic congestion and injury and primary hepatic insult from NAFLD.

Given these high rates of comorbid conditions, it is relevant that current therapeutic interventions used as first line therapy for these diseases have shown benefit from both a cardiac and hepatic perspective. Caloric restriction and aerobic exercise training additively improve exercise capacity in HFpEF, representing two of the only evidence-based therapies for the condition^29^. Similarly, structured nutrition and exercise interventions in NAFLD patients have successfully achieved weight loss is associated with improvements in degree of hepatic steatosis, inflammation and fibrosis^30^. Therefore, metabolic fitness programs could be designed to target both HFpEF and NAFLD concurrently^31^. As new treatments for NAFLD become available, the effect of these treatments on myocardial metabolism, structure and function should be investigated given the possibility of shared pathophysiologic mechanisms.

There are several limitations to our study that are important to highlight. First, the small sample size limits our ability to evaluate in detail parameters that might impact disease severity. Future studies with larger cohorts would have more power to evaluate correlations between more detailed ECHO parameters with prevalence and severity of NAFLD. Second, while patients were enrolled prospectively, NAFLD data were reviewed retrospectively. Such chart review has inherent limitations in terms of misclassification bias and missing data. However, we used previously validated methods to determine the presence of NAFLD and its severity. Both HFpEF and NAFLD disease classifications were made by experts in these disease states which represents a strength of the study as these entities can be difficult to assess. A combination of different diagnostic approaches is currently used in clinical practice for both NAFLD and HFpEF. The methods used in this paper reflect current clinical practice and society guidelines for making these diagnoses, though we acknowledge the limitation these place in terms of reproducibility of our study. Data capture in our electronic health system is also quite robust which is another strength of this study given the extent of characterization available for this cohort of patients. There is potential concern that patients with more advanced disease are those who underwent imaging, but analysis of patient characteristics by imaging status only noted differences in baseline anemia, lower RVSP, lower BNP and lower total cholesterol compared to those without imaging. As such, if anything this suggests that the imaging group may be lower risk for more advanced disease.

In conclusion, in this study of well characterized HFpEF patients, we noted a significantly higher prevalence of NAFLD (50%) compared to the general population and found that the majority of patients with NAFLD had evidence of advanced hepatic fibrosis. Presence of diabetes and obesity were most strongly correlated with NAFLD, whereas older individuals with these metabolic risk factors appeared to have highest rates of more advanced liver disease. Interestingly, no specific echocardiography parameters including right ventricular function or diastolic dysfunction were independently associated with more advanced liver disease. This questions the common assumption that cirrhosis in the setting of heart failure is from hepatic congestion alone^28^. Taken together, our data further adds to the discussion in terms of role for evaluation for underlying liver disease in patients with multiple cardio-metabolic risk factors as many of these patients already have advanced liver disease and liver disease stage has been shown to be linked with higher risk of adverse cardiac outcomes. As cost-effective screening and risk stratification methods for NAFLD continue to be refined, clinicians will be better able to tailor evaluation to at risk patients including those with HFpEF. In the interim, continued optimization of obesity and metabolic conditions remain a mainstay of therapy for both NAFLD and HFpEF.

## Supplementary information

Supplementary Information.
