# Supplementary material for: Prevalence and staging of non-alcoholic fatty liver disease among patients with heart failure with preserved ejection fraction
Source: Sci Rep. 2020 Jul 24;10:12440. doi: 10.1038/s41598-020-69013-y (PMC7381602; doi:10.1038/s41598-020-69013-y)
Supplement: Supplementary file 1 — Supplementary Information. [file 41598_2020_69013_MOESM1_ESM.pdf]

Prevalence and Staging of Non-Alcoholic Fatty Liver Disease Among Patients with Heart Failure with Preserved Ejection Fraction

Alexandria Miller MD* (2), Jennifer McNamara (3), Scott L. Hummel MD, MS (3, 4), Matthew C. Konerman MD (3), Monica A. Tincopa MD MSc (1)

(1) Division of Gastroenterology and Hepatology, Department of Internal Medicine, University of Michigan, Ann Arbor, MI
(2) Department of Internal Medicine, University of Michigan, Ann Arbor, MI
(3) Division of Cardiovascular Medicine, Department of Internal Medicine, University of Michigan, Ann Arbor, MI
(4) Ann Arbor Veterans Affairs Health System, Ann Arbor, MI

Corresponding Author: Monica A Tincopa, MD MSc**;** University of Michigan, 1500 East Medical Center Drive, Ann Arbor, MI 48109; tincopa@med.umich.edu

Supplemental Table 1. Multivariable Correlated of NAFLD Among Overall Cohort of HFpEF Patients

Variable	Odds Ratio	95% CI	P Value
Age (per year)	0.99	0.96 -1.03	0.90
Female Gender	1.46	0.65 – 3.27	0.35
BMI (per kg/m^2)	1.04	0.99 – 1.09	0.05
Diabetes Mellitus	1.77	0.81 – 3.89	0.14
LDL	0.98	0.97 – 0.99	**0.04**
Severe HF	1.63	0.71 – 3.71	0.24

Supplemental Table 2. Baseline characteristics of patients with and without abdominal imaging

Variable	Overall (n = 181)	Imaging (n = 96)	No Imaging (n = 85)	P Value
**Demographics**				
Age	70 (59 – 77)	69 (58 – 76)	71 (59 – 78)	0.2329
Female Gender	75 (41%)	39 (41%)	36 (42%)	0.8138
Weight (kg)	95.7 (79.4 – 115)	99.2 (80.6 – 114)	93.0 (78.7 – 114.9)	0.4462
BMI (kg/m^2)	35.1 (28.2 – 42.1)	35.1 (27.8 – 41.4)	34.9 (28.8 – 42.2)	0.8746
**Clinical Characteristics**				
Hypertension	147 (81%)	78 (81%)	69 (81%)	0.9899
Coronary Artery Disease	75 (41%)	39 (41%)	36 (42%)	0.8138
Diabetes Mellitus	87 (48%)	49 (51%)	38 (45%)	0.3945
Obesity (BMI > 30 kg/m^2)	125 (69%)	66 (69%)	59 (69%)	0.9234
Atrial Fibrillation	87 (48%)	44 (46%)	43 (50%)	0.5228
Dyslipidemia	119 (66%)	66 (69%)	53 (62%)	0.3654
Statin Use	123 (68%)	67 (70%)	56 (66%)	0.5738
CKD (GFR < 60 mL/min)	96 (53%)	50 (52%)	46 (54%)	0.7843
Anemia	83 (46%)	51 (53%)	32 (38%)	**0.0370**
**Heart Failure Severity**				
NYHA Class<br>I-II<br>III-IV	n = 176<br>69 (39%)<br>107 (61%)	n = 95<br>33 (35%)<br>62 (65%)	n = 81<br>36 (44%)<br>45 (56%)	0.1886
BNP (pg/mL)	117 (52.0 – 260)	102 (32.0 – 199)	165 (63.5 – 365)	**0.0054**
**Echocardiography**				
Ejection Fraction (%, n = 172)	60 (60 – 65)	60 (60 – 65)	60 (60 – 65)	0.3253
IVS (mm, n = 160)	10 (9.0 -12)	10 (9 -12)	10 (9 – 13)	0.6623
Left Atrial Diameter (mm, n = 168)	46 (40 – 52)	45 (39 – 50)	47 (42 – 54)	0.0757
RVSP (mm Hg, n = 116)	47 (35 – 58)	42 (34 – 54)	52 (37 – 61)	**0.0281**
E/A Ratio (n = 104)	1.2 (0.8 – 1.9)	1.1 (0.8 – 1.8)	1.3 (0.9 – 2.0)	0.2288
Diastolic Dysfunction (n = 91)<br>Grade 0-1 or indeterminate<br>Grade ≥ 2	 53 (58%)<br>38 (42%)	n = 49<br>31 (63%)<br>18 (37%)	n = 42<br>22 (52%)<br>20 (48%)	0.2939
RV Systolic Dysfunction (n = 166)	23 (14%)	9 (10%)	14 (18%)	0.1507
**Metabolic Evaluation**				
Total Cholesterol (mg/dL, n = 156)	156 (124 – 184)	150 (122 – 173)	167 (127 – 197)	**0.0361**
Triglycerides (mg/dL, n = 156)	124 (88 – 170)	106 (82 – 158)	136 (98 – 178)	0.0945
HDL (mg/dL, n = 156)	46 (38 – 59)	45 (36 – 54)	46 (40 – 60)	0.3851
LDL (mg/dL, n = 156)	76 (60-97)	71 (55 – 90)	84 (69 – 106)	**0.0157**
A1c (%, n = 131)	6.2 (5.7 – 7.6)	6.3 (5.7 – 7.6)	6.2 (5.8 – 7.6)	0.8701
**Hepatic Evaluation**				
Platelets (K/uL, n = 178)	215 (176 - 268)	216 (181 – 272)	214 (168 – 264)	0.6716
AST (IU/L, n = 179)	26 (20 – 31)	26 (20 – 32)	26 (20 – 31)	0.8170
ALT (IU/L, n = 179)	23 (17 – 32)	23 (17 – 30)	24 (17 – 32)	0.4831

INR (n = 160)	1.1 (1.0 – 1.4)	1.1 (1.0 – 1.3)	1.1 (1.0 – 1.5)	0.9154
Total Bilirubin (mg/dL, n = 178)	0.5 (0.4 – 0.8)	0.5 (0.4 – 0.8)	0.5 (0.3 – 0.8)	0.8589
Albumin (g/dL, n = 178)	4.1 (3.9 – 4.4)	4.2 (3.8 – 4.4)	4.1 (3.9 – 4.4)	0.9918
FIB-4 (n = 160)	1.6 (1.2 – 2.5)	1.6 (1.1 – 2.3)	1.8 (1.2 – 2.7)	0.5902
NFS (n = 159)	0.33 (-0.72 – 1.4)	0.32 (-0.82 – 1.4)	0.34 (-0.50 – 1.4)	0.7316
*Continuous variables presented as median with interquartile range.				
